# On Matrix Representation of Extension Field GF(*p^L^*) and Its Application in Vector Linear Network Coding

**DOI:** 10.3390/e26100822

**Published:** 2024-09-26

**Authors:** Hanqi Tang, Heping Liu, Sheng Jin, Wenli Liu, Qifu Sun

**Affiliations:** School of Computer and Communication Engineering, University of Science and Technology Beijing, Beijing 100083, China; tanghanqi1009@ustb.edu.cn (H.T.); 13121778655@163.com (H.L.); 13735677295@163.com (S.J.); lilyaccount00@163.com (W.L.)

**Keywords:** vector linear network coding, matrix representation, finite field

## Abstract

For a finite field GF($p^{L}$) with prime *p* and L>1, one of the standard representations is L×L matrices over GF(*p*) so that the arithmetic of GF($p^{L}$) can be realized by the arithmetic among these matrices over GF(*p*). Based on the matrix representation of GF($p^{L}$), a conventional linear network coding scheme over GF($p^{L}$) can be transformed to an *L*-dimensional vector LNC scheme over GF(*p*). Recently, a few real implementations of coding schemes over GF($2^{L}$), such as the Reed–Solomon (RS) codes in the ISA-L library and the Cauchy-RS codes in the Longhair library, are built upon the classical result to achieve matrix representation, which focuses more on the structure of every individual matrix but does not shed light on the inherent correlation among matrices which corresponds to different elements. In this paper, we first generalize this classical result from over GF($2^{L})$ to over GF($p^{L}$) and paraphrase it from the perspective of matrices with different powers to make the inherent correlation among these matrices more transparent. Moreover, motivated by this correlation, we can devise a lookup table to pre-store the matrix representation with a smaller size than the one utilized in current implementations. In addition, this correlation also implies useful theoretical results which can be adopted to further demonstrate the advantages of binary matrix representation in vector LNC. In the following part of this paper, we focus on the study of vector LNC and investigate the applications of matrix representation related to the aspects of random and deterministic vector LNC.

## 1. Introduction

The finite fields GF($p^{L}$) with a prime of *p* and an integer of L≥1 have been widely used in modern information coding, information processing, cryptography, and so on. Specifically, in the study of *linear network coding* (LNC), conventional LNC [1] transmits data symbols along the edges over GF($p^{L}$), and every outgoing edge of a node *v* transmits a data symbol that is a GF($p^{L}$)-linear combination of the incoming data symbols to *v*. A general LNC framework called *vector* LNC [2] models the *data unit* transmitted along every edge as an *L*-dimensional vector of data symbols over GF(*p*). Correspondingly, the coding operations at *v* involve GF(*p*)-linear combinations of all data symbols in incoming data unit vectors and are naturally represented by L×L matrices over GF(*p*).

Recently, many works [3,4,5,6,7] have shown that vector LNC has the potential to reduce extra coding overheads in networks relative to conventional LNC. In order to achieve vector LNC, a *matrix representation* of GF($p^{L}$) [8] is L×L matrices over GF(*p*) so that the arithmetic of GF($p^{L}$) can be realized by the arithmetic among these matrices over GF(*p*). Based on the matrix representation of GF($p^{L}$), a conventional LNC scheme over GF($p^{L}$) can be transformed to an *L*-dimensional vector LNC scheme over GF(*p*). In addition to the theory of LNC, many existing implementations of linear codes, such as the Cauchy-RS codes in the Longhair library [9] and the RS codes in the Jerasure library [10,11] and the latest release of the ISA-L library [12], also practically achieve arithmetic over GF($2^{L}$) using matrix representation.

In order to achieve the matrix representation of GF($p^{L}$), a classical result obtained in [13] relies on polynomial multiplications to describe the corresponding matrix of an element over GF($2^{L}$). A number of current implementations and studies (see, e.g., [9,10,11,12,13,14,15]) utilize such a characterization to achieve the matrix representation of GF($2^{L}$). However, the characterization in the present form focuses more on the structure of every individual matrix and does not shed light on the inherent correlation between matrices that corresponds to different elements. As a result, in the aforementioned existing implementations, the corresponding binary matrix is either independently computed on demand or fully stored in a lookup table as an L×L matrix over GF(2) in advance.

In the first part of this paper, we shall generalize the characterization of matrix representation from over GF($2^{L})$ to over GF($p^{L}$) and paraphrase it from the perspective of matrices with different powers so that the inherent correlation among these matrices will become more transparent. More importantly, this correlation motivates us to devise a lookup table to pre-store the matrix representation with a smaller size. Specifically, compared to the one adopted in the latest release of the ISA-L library [12], the table size is reduced by a factor of 1/L. Additionally, this correlation also implies useful theoretical results that can be adopted to further demonstrate the advantages of binary matrix representation in vector LNC. In the second part, we focus on the study of vector LNC and show the applications of matrix representation related to the aspects of random and deterministic coding. In random coding, we theoretically analyze the coding complexity of conventional and vector LNC via matrix representation under the same alphabet size $2^{L}$. The  comparison results show that vector LNC via matrix representation can reduce at least half of the coding complexity to achieve multiplications. Then, in deterministic LNC, we focus on the special choice of coding operations that can be efficiently implemented. In particular, we illustrate that the choice of primitive polynomial can influence the distributions of matrices with different numbers of non-zero entries and propose an algorithm to obtain a set of sparse matrices that can be good candidates for the coefficients of a practical LNC scheme.

This paper is structured as follows. Section 2 reviews the mathematical fundamentals of representations to an extension field GF($p^{L}$). Section 3 paraphrases the matrix representation from the perspective of matrices in different powers and then devises a lookup table to pre-store the matrix representation with a smaller size. Section 4 focuses on the study of vector LNC and shows the applications of matrix representation related to the aspects of random and deterministic coding. Section 5 summarizes this paper.

**Notation.** In this paper, every bold symbol represents a vector or a matrix. In particular, $I_{L}$ refers to the identity matrix of size *L*, and **0**, **1**, respectively, represent an all-zero or all-one matrix, whose size, if not explicitly explained, can be inferred in the context.

## 2. Preliminaries

In this section, we review three different approaches to express an extension field GF($p^{L}$) with $p^{L}$ elements, where *p* is a prime. The first approach is the standard *polynomial representation*. Let p(x) denote an irreducible polynomial of degree *L* over GF(*p*) and α be a root of p(x). Every element of GF($p^{L}$) can be uniquely expressed as a polynomial in α over GF(*p*) with a degree less than *L*, and $\left\{1,\alpha ,\alpha^{2},\ldots ,\alpha^{L-1}\right\}$ forms a basis GF($p^{L}$) over GF(*p*). In particular, every $\beta \in \mathrm{GF}(p^{L})$ can be uniquely represented in the form of $\sum_{l=0}^{L-1}v_{l}\alpha^{l}$ with $v_{l}\in \mathrm{GF}\left(p\right)$. In the polynomial representation, the element $\beta =\sum_{l=0}^{L-1}v_{l}\alpha^{l}$ is expressed as the *L*-dimensional *representative vector* $v_{\beta }={\left[v_{0}\;v_{1}\;\ldots \;v_{L-1}\right]}_{\beta }$ over GF(*p*). In order to further simplify this expression, $v_{\beta }$ can be written as the integer $0\leq d_{\beta }^{\mathrm{poly}}\leq p^{L}-1$ such that

(1) $$d_{\beta }^{\mathrm{poly}}=\sum_{l=0}^{L-1}p^{l}{\hat{v}}_{l},$$

where $0\leq {\hat{v}}_{l}<p$ is the integer representation of $v_{l}$, that is, $\sum_{i=1}^{{\hat{v}}_{l}}1=v_{l}$ where 1 is to be the multiplicative unit of GF(*p*).

The second approach is called the *generator representation*, which further requires p(x) to be a primitive polynomial such that α is a primitive element, and  all $p^{L}-1$ non-zero elements in GF($p^{L}$) can be generated as $\alpha^{0},\alpha^{1},\alpha^{2},\ldots ,\alpha^{p^{L}-2}$. Thus, every non-zero $\beta \in \mathrm{GF}(p^{L})\setminus 0$ is uniquely expressed as the integer $0\leq d_{\beta }^{\mathrm{gen}}\leq p^{L}-2$ subject to

(2) $$\beta =\alpha^{d_{\beta }^{\mathrm{gen}}}.$$

The *polynomial representation* clearly specifies the additive structure of GF($p^{L}$) as a vector space or a quotient ring of polynomials over GF(*p*) while leaving the multiplicative structure hard to determine. Meanwhile, the *generator representation* explicitly illustrates the cyclic multiplicative group structure of $\mathrm{GF}\left(p^{L}\right)\setminus \left\{0\right\}$ without clearly demonstrating the additive structure. It turns out that the addition operation and its inverse in $\mathrm{GF}(p^{L})$ are easy to implement based on the *polynomial representation*, while the multiplicative operations and its inverse in $\mathrm{GF}(p^{L})$ are easy to be implement based on the *generator representation*. In particular, for $\beta_{1},\beta_{2}\in \mathrm{GF}\left(p^{L}\right)$,

(3) $$d_{\beta_{1}+\beta_{2}}^{\mathrm{poly}}=d_{\beta_{1}}^{\mathrm{poly}}⊕d_{\beta_{2}}^{\mathrm{poly}}\;\;\mathrm{or}\;\mathrm{equivalently}\;\;v_{\beta_{1}+\beta_{2}}=v_{\beta_{1}}+v_{\beta_{2}}$$

(4) $$d_{\beta_{1}\beta_{2}}^{\mathrm{gen}}=\left(d_{\beta_{1}}^{\mathrm{gen}}+d_{\beta_{2}}^{\mathrm{gen}}\right)\;\mathrm{mod}\;p^{L}-1,$$

where the operation ⊕ between two integers $d_{\beta_{1}}^{\mathrm{poly}}$ and $d_{\beta_{2}}^{\mathrm{poly}}$ means the component-wise *p*-ary addition $v_{1}+v_{2}$ between the *p*-ary expression $v_{1}$, $v_{2}$ of them. This is the key reason that in practice both representations are always adopted interchangeably when conducting operations in GF($p^{L}$).

Unfortunately, except for some special $\beta \in \mathrm{GF}(p^{L})$, such as $\alpha^{l}$, 0≤l<L, there is not a straightforward way to establish the mapping between $d_{\beta }^{\mathrm{poly}}$ and $d_{\beta }^{\mathrm{gen}}$ without computation, and a built-in lookup table is always adopted in practice to establish the mapping between two types of representations. For instance, Table 1 lists the mapping between $d_{\beta }^{\mathrm{poly}}$ and $d_{\beta }^{\mathrm{gen}}$ for non-zero elements β in $\mathrm{GF}(2^{3})$ with $p\left(x\right)=x^{4}+x+1$.

By convention, elements β in GF($p^{L}$) are represented as $d_{\beta }^{\mathrm{poly}}$. It takes *L p*-ary additions to compute $d_{\beta_{1}+\beta_{2}}=d_{\beta_{1}}^{\mathrm{poly}}⊕d_{\beta_{1}}^{\mathrm{poly}}$. Based on the lookup table, it takes 3 lookups (which, respectively, map $d_{\beta_{1}}^{\mathrm{poly}}$, $d_{\beta_{2}}^{\mathrm{poly}}$ to $d_{\beta_{1}}^{\mathrm{gen}}$, $d_{\beta_{2}}^{\mathrm{gen}}$ and $d_{\beta_{1}\beta_{2}}^{\mathrm{gen}}$ to $d_{\beta_{1}\beta_{2}}^{\mathrm{poly}}$), 1 integer addition, and at most 1 modulo $p^{L}-1$ operation to compute $d_{\beta_{1}\beta_{2}}^{\mathrm{poly}}=d_{\beta_{1}}^{\mathrm{poly}}d_{\beta_{2}}^{\mathrm{poly}}$. Meanwhile, it is worthwhile to note that the calculation of $d_{\beta_{1}\beta_{2}}^{\mathrm{poly}}=d_{\beta_{1}}^{\mathrm{poly}}d_{\beta_{2}}^{\mathrm{poly}}$ without the table follows the multiplication of polynomials $f_{1}\left(x\right)$ and $f_{2}\left(x\right)$ with coefficient vectors $v_{\beta_{1}}$ and $v_{\beta_{2}}$, respectively, and finally falls into

(5) $$f_{1}\left(x\right)f_{2}\left(x\right)\;\mathrm{modulo}\;p\left(x\right),$$

where the computational complexity compared with the following matrix representation will be fully discussed in Section 4.

The third approach, which is the focus of this paper, is given by means of matrices called the *matrix representation* [8]. Let C be the L×L companion matrix of an irreducible polynomial p(x) of degree *L* over GF(*p*). In particular, if $p\left(x\right)=a_{0}+a_{1}x+a_{2}x^{2}+\ldots +a_{L-1}x^{L-1}+x^{L}$ with $a_{0},a_{1},\ldots ,a_{L-1}\in \mathrm{GF}\left(p\right)$,   

(6) $$C={\left[\begin{array}{cc} 0 & -a_{0}\\ I_{L-1} & \begin{array}{c} -a_{1}\\ \ldots \\ -a_{L-1} \end{array} \end{array}\right]}_{L\times L}.$$

It can be easily verified that p(x) is the characteristic polynomial of C, and  according to the Cayley–Hamilton theorem, p(C)=0. As a result, $\left\{I_{L},C,C^{2},\cdots ,C^{L-1}\right\}$ forms a basis of GF($p^{L}$) over GF(*p*), and for every $\beta \in \mathrm{GF}(p^{L})$ with the representative vector $v_{\beta }={\left[v_{0}\;v_{1}\;\ldots \;v_{L-1}\right]}^{T}$ based on the polynomial representation, the matrix representation M(β) of β is defined as

(7) $$M\left(\beta \right)=\sum_{i=0}^{L-1}v_{i}C^{i}.$$

If the considered p(x) further qualifies as a primitive polynomial, then similar to the role of the primitive element α defined above, C is also a multiplicative generator of all non-zero elements in GF($p^{L}$), that is, $M\left(\alpha^{i}\right)=C^{i}$ for all $0\leq i\leq p^{L}-2$. One advantage for the matrix representation is that all operations in GF($p^{L}$) can be realized by matrix operations over GF(*p*) among the matrices in C such that there is no need to interchange between the polynomial and the generator representations in performing field operations. For more detailed discussions of representation of an extension field, please refer to [16].

Based on the polynomial representation and generator representation, even though the arithmetic over GF($p^{L}$) can be efficiently realized by (3), (4) and a lookup table, it requires two different types of calculation systems, i.e., one over GF(*p*) and the other over integers. This hinders the deployment practicality in applications with resource-constrained edge devices, such as in ad hoc networks or Internet of Things applications. In comparison, the matrix representation of GF($p^{L}$) interprets the arithmetic of GF($p^{L}$) solely over the arithmetic over GF(*p*), so it is also a good candidate for realization of the efficient implementation of linear codes over GF($p^{L}$) such as in [9,10,11,12,13].

## 3. Useful Characterization of the Matrix Representation

Let p(x) be a defined irreducible polynomial over GF(*p*) of degree *L* and let $\alpha \in \mathrm{GF}(p^{L})$ be a root of p(x). When p=2, a useful characterization of the matrix representation M(β) of $\beta \in \mathrm{GF}(p^{L})$ (with respect to p(x)) can be deduced based on the following classical result obtained in Construction 4.1 and Lemma 4.2 of [13]: For 1≤j≤L, the *j*th column in M(β) is equal to the binary expression of $\alpha^{j-1}\beta$ based on the polynomial representation. A number of implementations and studies (see, e.g.,  [9,10,11,12,13,14,15]) of linear codes utilize such characterization to achieve the matrix representation of GF($2^{L}$). However, the characterization in the present form relies on polynomial multiplications and focuses more on the structure of every individual M(β). It does not explicitly shed light on the inherent correlation among M(β) of different $\beta \in \mathrm{GF}(2^{L})$. It turns out that in existing implementations, such as the Cauchy-RS codes in the Longhair library [9] and the RS codes in the Jerasure library [10,11], and the latest release of ISA-L library [12], M(β) is either independently computed on demand or fully stored in a lookup table as an L×L matrix over GF(2) in advance.

In this section, we shall generalize the characterization of matrix representation from over GF($2^{L})$ to over GF($p^{L}$) and paraphrase it based on the interplay with the generator representation instead of the conventional polynomial representation so that the correlation among M(β) of different $\beta \in \mathrm{GF}(p^{L})$ will become more transparent. From now on, we assume that p(x) is further qualified to be a primitive polynomial such that α is a primitive element in $\mathrm{GF}(p^{L})$. For simplicity, let $v_{i}$, $0\leq i\leq p^{L}-2$, denote the representative (column) vector of $\alpha^{i}$ based on the polynomial representation. Then, the following theorem asserts that the matrix representation $M\left(\alpha^{i}\right)=C^{i}$ consists of *L* representative vectors with consecutive subscripts.

**Theorem** **1.** *For $0\leq i\leq p^{L}-2$, the matrix representation $M\left(\alpha^{i}\right)=C^{i}$ can be written as follows:*

(8) $$C^{i}=\left[\begin{array}{cccc} v_{i} & v_{i+1} & \cdots & v_{i+L-1} \end{array}\right].$$

*As $C^{p^{L}-1}=I_{L}$, we omit the modulo-$\left(p^{L}-1\right)$ expressions on the exponent of C and subscript of v throughout this paper for brevity.*

**Proof.**  First, the matrix $C^{i}$ can be characterized by multiplication iterations based on (6) as follows. When 2≤i≤L,

(9) $$C^{i}=\left[\begin{array}{cc} U & \begin{array}{ccccc} -a_{0} & p_{0}^{\left(1\right)} & p_{0}^{\left(2\right)} & \cdots & p_{0}^{\left(i-1\right)}\\ -a_{1} & p_{1}^{\left(1\right)} & p_{1}^{\left(2\right)} & \cdots & p_{1}^{\left(i-1\right)}\\ \vdots & \vdots & \vdots & \ddots & \vdots \\ -a_{L-2} & p_{L-2}^{\left(1\right)} & p_{L-2}^{\left(2\right)} & \cdots & p_{L-2}^{\left(i-1\right)}\\ -a_{L-1} & p_{L-1}^{\left(1\right)} & p_{L-1}^{\left(2\right)} & \cdots & p_{L-1}^{\left(i-1\right)} \end{array} \end{array}\right],$$

where L×(L−i) matrix $U=\begin{array}{c} \left[\begin{array}{c} 0\\ I_{L-i} \end{array}\right] \end{array}$. Further, when $L+1\leq i\leq p^{L}-2$,

(10) $$C^{i}=\left[\begin{array}{cccc} p_{0}^{\left(i-L\right)} & p_{0}^{\left(i-L+1\right)} & \cdots & p_{0}^{\left(i-1\right)}\\ p_{1}^{\left(i-L\right)} & p_{1}^{\left(i-L+1\right)} & \cdots & p_{1}^{\left(i-1\right)}\\ \vdots & \vdots & \ddots & \vdots \\ p_{L-2}^{\left(i-L\right)} & p_{L-2}^{\left(i-L+1\right)} & \cdots & p_{L-2}^{\left(i-1\right)}\\ p_{L-1}^{\left(i-L\right)} & p_{L-1}^{\left(i-L+1\right)} & \cdots & p_{L-1}^{\left(i-1\right)} \end{array}\right].$$

The entries in (9) and (10) iteratively qualify

(11) $$p_{0}^{\left(1\right)}=-a_{0}a_{L-1},\;p_{j}^{\left(1\right)}=a_{j-1}-a_{j}a_{L-1},1\leq j\leq L-1$$

and

(12) $$\begin{array}{c} \begin{array}{cc} p_{0}^{\left(k\right)} & =-a_{0}p_{L-1}^{\left(k-1\right)},\\ \;p_{j}^{\left(k\right)} & =p_{j-1}^{\left(k-1\right)}-a_{j}p_{L-1}^{\left(k-1\right)},1\leq j\leq L-1,2\leq k\leq i-1. \end{array} \end{array}$$

When i=0, it can be easily checked that each vector in $\left\{v_{0},v_{1},v_{2},\ldots ,v_{L-1}\right\}$ is a unit vector such that the only non-zero entry 1 of $v_{i}$ locates at (i+1)th row. Therefore, $C^{0}=I_{L}=\left[v_{0},v_{1},v_{2},\ldots ,v_{L-1}\right]$, and (8) holds. When i=1, consider $v_{L}$ with p(C)=0, i.e.,

(13) $$a_{0}I_{L}+a_{1}C+a_{2}C^{2}+\ldots +a_{L-1}C^{L-1}+C^{L}=0.$$

Obviously, $v_{L}={\left[\begin{array}{cccc} -a_{0} & -a_{1} & \ldots & -a_{L-1} \end{array}\right]}^{T}$ and (8) holds.

Assume when i=m, (8) holds, i.e., $C^{m}=\left[\begin{array}{cccc} v_{m} & v_{m+1} & \cdots & v_{m+L-1} \end{array}\right]$. The *L*th column vector ${\left[\begin{array}{cccc} p_{0}^{\left(m-1\right)} & p_{1}^{\left(m-1\right)} & \ldots & p_{L-1}^{\left(m-1\right)} \end{array}\right]}^{T}$ of $C^{m}$ based on (9) corresponds to the representative vector of $C^{m+L-1}$, that is, the matrix $C^{m+L-1}$ is equal to

(14) $$p_{0}^{\left(m-1\right)}I_{L}+p_{1}^{\left(m-1\right)}C+\ldots +p_{L-2}^{\left(m-1\right)}C^{L-2}+p_{L-1}^{\left(m-1\right)}C^{L-1}$$

It remains to prove, by induction, that $C^{m+1}=\left[\begin{array}{cccc} v_{m+1} & v_{m+2} & \cdots & v_{m+L} \end{array}\right].$ As the column vectors indexed from 1th to (L−1)th of matrix $C^{m+1}$ are exactly same as the ones indexed from 2th to *L*th of $C^{m}$, it suffices to show that the *L*th column vector of $C^{m+1}$ corresponds to $v_{m+L}$. The following is based on (13) and (14):

$$\begin{array}{cc} C^{m+L} & =p_{0}^{\left(m-1\right)}C+p_{1}^{\left(m-1\right)}C^{2}+\ldots +p_{L-2}^{\left(m-1\right)}C^{L-1}+p_{L-1}^{\left(m-1\right)}C^{L}\\ \; & =p_{0}^{\left(m-1\right)}C+p_{1}^{\left(m-1\right)}C^{2}+\ldots +p_{L-2}^{\left(m-1\right)}C^{L-1}-p_{L-1}^{\left(m-1\right)}\left(a_{0}I_{L}+\ldots +a_{L-1}C^{L-1}\right)\\ \; & =-a_{0}p_{L-1}^{\left(m-1\right)}I_{L}+\left(p_{0}^{\left(m-1\right)}-a_{1}p_{L-1}^{\left(m-1\right)}\right)C+\ldots +\left(p_{L-2}^{\left(m-1\right)}-a_{L-1}p_{L-1}^{\left(m-1\right)}\right)C^{L-1}. \end{array}$$

It can be easily checked that $p_{0}^{\left(m\right)}$ and $p_{j}^{\left(m\right)}$ with 1≤j≤L−1 in $C^{m+1}$ calculated by (12) exactly consist of the representative vector of $C^{m+L}$, i.e., $v_{m+L}$. This completes the proof. □

The above theorem draws an interesting conclusion that every non-zero matrix in C is composed of *L* representative vectors. Specifically, the first column vector of the matrix representation $C^{i}$ is the representative vector of $\alpha^{i}$, and its *j*th column vector, 1≤j≤L, corresponds to the representative vector of $\alpha^{i+j-1}$. For the case p=2, even though the above theorem is essentially same as Construction 4.1 and Lemma 4.2 in [13], its expression with the interplay of generator representation allows us to further devise a lookup table to pre-store the matrix representation with a smaller size.

In this table, we store $p^{L}$ representative vectors with table size $L\times p^{L}$ and arrange them based on the power order of α with $0\leq i\leq p^{L}-2$. Note that the first column of matrix $C^{i}$ can be indexed by vector $v_{i}$ or (i+1)th column in this table, and the remaining columns of $C^{i}$ can be obtained via subsequent L−1 column vectors based on Theorem 1. As a result, although this table only stores $p^{L}$ vectors, it contains the whole matrix representations of $\mathrm{GF}(p^{L})$ due to the inherent correlation among $C^{i}$. The following Example 1 shows the explicit lookup table of $\mathrm{GF}(2^{4})$ as an example.

**Example** **1.** *Consider the field GF($2^{4}$) and primitive polynomial $p\left(x\right)=1+x+x^{4}$ over GF(2). The  companion matrix C is written as follows:*

$$\begin{array}{c} C=\left[\begin{array}{cccc} 0 & 0 & 0 & 1\\ 1 & 0 & 0 & 1\\ 0 & 1 & 0 & 0\\ 0 & 0 & 1 & 0 \end{array}\right]. \end{array}$$

**Figure 1:**
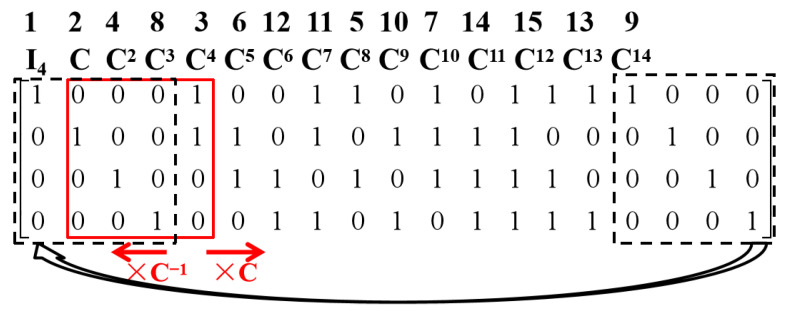
The lookup table to store the matrix representation $C^{i}$ with 0≤i≤14 for the field GF($2^{4}$) and primitive polynomial $p\left(x\right)=1+x+x^{4}$.

*Then, the lookup table to store matrix representation $C^{i}$ with 0≤i≤14 is shown in Figure 1. In this figure, the solid “window” that currently represents the matrix C can be slid to the right or left to generate $C^{i}$ with different i; meanwhile, the  dashed box shows the cyclic property based on cyclic group $\left\{I_{4}=C^{15},C,C^{2},\cdots ,C^{14}\right\}$.*

Recall that in the lookup table of the matrix representation adopted in the latest release of the ISA-L library [12], the matrix representation of every element in $\mathrm{GF}(p^{L})$ needs to be stored, so a total of $L^{2}p^{L}$ *p*-ary elements need to be pre-stored. Compared with that, only an $L\times p^{L}$ *p*-ary matrix needs to be stored in the new lookup table, so the table size is reduced by a factor of 1/L. Moreover, Theorem 1 implies the following useful corollaries of the matrix representation $C=\{0,C^{0},C^{1},\cdots ,C^{p^{L}-2}\}$ of $\mathrm{GF}(p^{L})$.

**Corollary** **1.** *Every vector in the vector space GF(${p)}^{L}$ exactly occurs L times as a column vector in matrices of C.*

**Proof.**  As $\left\{I_{L},C,C^{2},\cdots ,C^{L-1}\right\}$ forms a polynomial basis of GF($p^{L}$) over GF(*p*), the representative vectors of matrices in C are distinct. Consider a function $f:\left\{C^{i}\right\}\to \left\{v_{i}\right\}$. It can be easily checked that *f* is bijective, and $v_{i}$ exactly corresponds to the *j*th column vector of $C^{i-j+1}$ with 1≤j≤L. The zero vector of length *L* simply occurs *L* times in L×L matrix 0.    □

**Corollary** **2.** *For every GF($p^{L}$), regardless of the choice of the primitive polynomial p(x), the total number of zero entries in C remains unchanged as $L^{2}p^{L-1}$.*

The above two corollaries will be adopted to further demonstrate the advantages of binary matrix representation in vector LNC with C.

## 4. Applications of Matrix Representation in Vector LNC

In this section, we focus on the study of vector LNC with binary matrices C and show the applications of matrix representation related to the aspects of random and deterministic coding.

### 4.1. Computational Complexity Comparison in Random LNC

Herein, the coding coefficients of random LNC are randomly selected from GF($2^{L}$), which can provide a distributed and asymptotically optimal approach for information transmission, especially in unreliable or topologically unknown networks, such as wireless broadcast networks [17] or ad hoc network [18]. Recall that in polynomial and generator representations, the multiplication over GF($2^{L}$) based on a lookup table requires two different types of calculation systems, so this table may not be utilized in resource-constrained edge devices. Therefore, under the same alphabet size $2^{L}$, we first theoretically compare the random coding complexity between conventional LNC over $\left\{\beta =\sum_{l=0}^{L-1}v_{l}\alpha^{l}\right\}$ and vector LNC over C without lookup table, from the perspective of required binary operations.

To keep the same benchmark for complexity comparison, we adopt the following assumptions.

- We assume that an all-1 binary vector m as information will multiply $2^{L}-1$ non-zero coding coefficients selected from $\left\{\beta =\sum_{l=0}^{L-1}v_{l}\alpha^{l}\right\}$ and C, which can be simulated as encoding process. The complexity is the total number of binary operations that $2^{L}-1$ multiplications take.
- We shall ignore the complexity of a shifting or permutation operation on the binary vector m, which can be efficiently implemented.
- We only consider the standard implementation of multiplication in GF($2^{L}$) by polynomial multiplication modulo and primitive polynomial $p\left(x\right)=a_{0}+a_{1}x+\cdots +a_{L-1}x^{L-1}+x^{L}$ with η non-zero $a_{i},0\leq i\leq L-1$, instead of considering other advanced techniques such as the FFT algorithm [19].

We first consider the encoding scheme with coefficients selected from $\left\{\beta =\sum_{l=0}^{L-1}v_{l}\alpha^{l}\right\}$. Assume that α is a root of p(x) and every element β in GF($2^{L}$) can be expressed as g(α), where g(x) represents a polynomial over GF(2) with a degree less than *L*. An all-1 binary information vector m can be expressed as $\alpha^{L-1}+\alpha^{L-2}+\cdots +\alpha^{2}+\alpha +1$. We can divide the whole encoding process into two parts: multiplication and addition. In the multiplication part, the complexity of shifting operations is ignored, and one polynomial $m\alpha^{i}$ in mg(α) will modulo p(x) *i* times and take iη binary operations. Because every $\alpha^{i},1\leq i\leq L-1$ occurs $2^{L-1}$ times among all g(α) in GF($2^{L}$), it will take $\sum_{1\leq i\leq L-1}i\eta \times 2^{L-1}$ binary operations to compute $m\alpha^{i}$. In the addition part, it takes (j−1)L binary operations to compute the additions between *j* binary vectors $m\alpha^{i}$ with distinct *i*. Note that the number of distinct g(α) with *j* non-zero terms is Lj in GF($2^{L}$). Therefore, the traverse of g(α) will take an extra ∑1≤j≤LLj×(j−1)L binary additions to compute mg(α). In total, the complexity of this scheme is shown as follows:

(15) ∑1≤i≤L−1i(η×2L−1+L×Li+1).

Next, we consider the encoding scheme with coefficients selected from C, whose complexity of encoding process depends on the total number of 1 in $C^{i}$ with $0\leq i\leq 2^{L}-2$. In this framework, it is worthwhile to note that every $C^{i}$ in C is full-rank and can extract a permutation matrix. Since the complexity of permutational operations is ignored, based on Proposition 2, the complexity of encoding process over C is shown as follows:

(16) $$L^{2}\times 2^{L-1}-L\times \left(2^{L}-1\right)=2^{L-1}\left(L^{2}-2L\right)+L.$$

For any primitive polynomial p(x), η≥2. With 3≤L≤12, Table 2 lists the *average* number of binary operations *per symbol* in two schemes. Specifically, every value calculated by Equation (15) and (16) has divided the alphabet size $2^{L}$, and we can find that in random coding, the vector LNC via matrix representation can theoretically reduce at least half of the coding complexity to achieve multiplications under the same alphabet size $2^{L}$.

### 4.2. The Special Choices of Binary p(x) and Sparse $C^{i}$

In addition to the random coding, a deterministic LNC where we pay a broader concern to reduce the computational complexity can also carefully design some special coding operations which can be efficiently implemented, such as circular shift [5,6] or permutation [7]. In this subsection, different from random choice of coefficients, we will carefully design the choices of binary primitive polynomial p(x) and sparse matrices $C^{i}$ in C based on the unveiled properties in Sec. III. We illustrate that the choice of p(x) can influence the distributions of matrices $C^{i}$ with different numbers of non-zero entries. Then, based on a proper p(x), an algorithm is proposed to obtain a subset of C, which contains a series of relatively sparse matrices in C.

When p=2, the entries in representative vectors based on Equation (11) and (12) will, respectively, degenerate as follows:

(17) $$\begin{array}{c} \begin{array}{cc} p_{0}^{\left(1\right)} & =a_{L-1},\\ p_{j}^{\left(1\right)} & =a_{j-1}+a_{j}a_{L-1}=a_{j-1}+a_{j}p_{0}^{\left(1\right)},1\leq j\leq L-1. \end{array} \end{array}$$

and

(18) $$\begin{array}{c} \begin{array}{cc} p_{0}^{\left(k\right)} & =p_{L-1}^{\left(k-1\right)},\\ p_{j}^{\left(k\right)} & =p_{j-1}^{\left(k-1\right)}+a_{j}p_{L-1}^{\left(k-1\right)}=p_{j-1}^{\left(k-1\right)}+a_{j}p_{0}^{\left(k\right)},1\leq j\leq L-1,2\leq k\leq i-1. \end{array} \end{array}$$

with $a_{0}$ must be 1 in p(x). Based on the above two equations, consider two adjacent representative vectors $v_{k-1}$ and $v_{k}$. When the last entry $p_{L-1}^{\left(k-1\right)}$ in $v_{k-1}$ is equal to 0, the entries in $v_{k}$ follow

$$\begin{array}{c} p_{0}^{\left(k\right)}=0,\;p_{j}^{\left(k\right)}=p_{j-1}^{\left(k-1\right)},1\leq j\leq L-1, \end{array}$$

which means that $v_{k}$ can be generated by downward circular shift to $v_{k-1}$. When the last entry $p_{L-1}^{\left(k-1\right)}$ equals 1, the entries in $v_{k}$ follow

$$\begin{array}{c} p_{0}^{\left(k\right)}=1,\;p_{j}^{\left(k\right)}=p_{j-1}^{\left(k-1\right)}+a_{j},1\leq j\leq L-1. \end{array}$$

Therefore, the difference between $v_{k-1}$ and $v_{k}$ in Hamming weight is no more than η−1, where η represents the number of non-zero $a_{i},0\leq i\leq L-1$ in primitive polynomial p(x).

Note that for the matrix representation of every GF($2^{L}$), the total number of 1 in C is always $L^{2}\times 2^{L-1}$ regardless of the choice of binary p(x). However, the value of η will influence the distributions of sparse matrices in C. Based on (17) and (18), we can intuitively deduce that with smaller η, the sparse matrices in C will be more concentrated distribution. Since the identity matrix $I_{L}$ with *L* non-zero entries is the sparsest full-rank matrix, we utilize Algorithm 1 to choose $2^{L-s}$ matrices in C, which can be good candidates as coding coefficients of a practical coding scheme over GF($2^{L}$).
**Algorithm 1** The choice of sparse matrices over GF($2^{L}$)**Initialize** *S* as an empty set of L×L binary matrix     S←0     $S\leftarrow I_{L}$     generate matrix C based on p(x)     generate matrix $C^{-1}$ based on Equation (18)     define matrix $\hat{C}=C$     define matrix ${\hat{C}}^{-1}=C^{-1}$     define integer s<L: the required size of $C_{s}$**for** $i=1:2^{L-s-1}-1$     $S\leftarrow \hat{C}$     $S\leftarrow {\hat{C}}^{-1}$     $\hat{C}=\hat{C}\times C$     ${\hat{C}}^{-1}={\hat{C}}^{-1}\times C^{-1}$     i=i+1**end****return** *S*

In Algorithm 1, the multiplications using C or $C^{-1}$ can be easily achieved by sliding the “window” right or left, respectively, as shown in Figure 1. Let $C_{s}$ denote this subset of C and the $2^{L-s}$ matrices in $C_{s}$ can be written as $\left\{0,I_{L},C^{i},C^{-i}\right\}$ with $1\leq i\leq 2^{L-s-1}-1$. Then, Table 3 lists the ratio of the total number of 1 between $C_{s}$ and C with s=1,2. We can find that the $2^{L-s}$ matrices, which are special choices using Algorithm 1, indeed contain less 1 than the other matrices in C.

**Figure 2:**
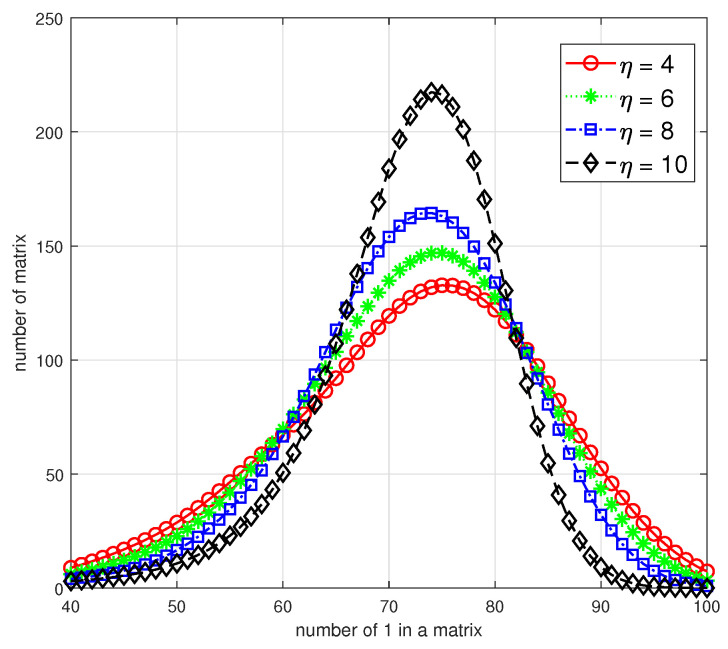
The distribution of sparse matrices in C with different η=4,6,8,10.

Moreover, in Figure 2, we numerically analyze the relationship between the number of 1 in each matrix and the corresponding number of matrices under the alphabet size $2^{12}$. For all candidates of binary primitive polynomials, we choose four representative p(x) with different η=4,6,8,10 and the specific polynomials are shown as follows:

$$\begin{array}{cc} p_{1}\left(x\right) & =x^{12}+x^{6}+x^{4}+x^{1}+1\\ p_{2}\left(x\right) & =x^{12}+x^{7}+x^{6}+x^{5}+x^{3}+x^{1}+1\\ p_{3}\left(x\right) & =x^{12}+x^{8}+x^{7}+x^{6}+x^{4}+x^{3}+x^{2}+x^{1}+1\\ p_{4}\left(x\right) & =x^{12}+x^{10}+x^{9}+x^{8}+x^{7}+x^{5}+x^{4}+x^{3}+x^{2}+x^{1}+1 \end{array}$$

As all the matrices in C are full-rank, the value range of the x-axis should be [12,132], and we restrict it to [40,100] to highlight the distributions. These four curves illustrate that with η increasing, the distribution variance of the number of 1 in a matrix will decrease, that is, the number of matrices with an average number of 1, i.e., 70–80, will increase and the number of relatively sparse or dense matrices will decrease. As a result, a smaller η of p(x) not only infers a more concentrated distribution but also more amounts for sparse matrices in C; then, we can select parameter *s* according to practical requirements and obtain $C_{s}$ using Algorithm 1.

## 5. Conclusions

Compared with the classical result, the paraphrase of matrix representation in this paper focuses more on inherent correlation among matrices and a lookup table to pre-store the matrix representation with a smaller size is devised. This work also identifies that the total number of non-zero entries in C is a constant number, which motivates us to demonstrate the advantages of binary matrix representation in vector LNC. In the applications of matrix representation, we first theoretically demonstrate the vector LNC via matrix representation can reduce at least half of the coding complexity compared with conventional one over GF($2^{L}$). Then, we illustrate the influence of η, i.e., the number of non-zero item in p(x), on the amounts and distributions of sparse matrices in C and propose an algorithm to obtain sparse matrices which can be good candidates as coding coefficients of a practical vector LNC scheme.

## Figures and Tables

**Table 1:** The mapping between $d_{\beta }^{\mathrm{poly}}$ and $d_{\beta }^{\mathrm{gen}}$ for non-zero β in $\mathrm{GF}(2^{4})$ with $p\left(x\right)=x^{4}+x+1$.

$d_{\beta }^{\mathrm{gen}}$	0	1	2	3	4	5	6	7	8	9	10	11	12	13	14
$d_{\beta }^{\mathrm{poly}}$	1	2	4	8	3	6	12	11	5	10	7	14	15	13	9

**Table 2:** Average number of binary operations per symbol with parameter η=2.

*L*	3	4	5	6	7	8	9	10	11	12
C	1.88	4.25	7.66	12.09	17.55	24.03	31.52	40.01	49.51	60.01
$\sum_{l=0}^{L-1}v_{l}\alpha^{l}$	4.88	10.25	17.66	27.09	38.55	52.03	67.52	85.01	104.51	126.01
rate	38.5%	41.5%	43.3%	44.6%	45.5%	46.2%	46.7%	47.1%	47.3%	47.6%

**Table 3:** Ratio of total numbers of 1 between $C_{s}$ and C.

*L*	p(x)	η	s=1	s=2
3	$X^{3}+X+1$	2	0.3056	0.0833
4	$X^{4}+X+1$	2	0.3438	0.1094
5	$X^{5}+X^{2}+1$	2	0.3800	0.1200
6	$X^{6}+X+1$	2	0.4410	0.1372
7	$X^{7}+X+1$	2	0.4585	0.1987
8	$X^{8}+X^{4}+X^{3}+X^{2}+1$	4	0.4635	0.2235
9	$X^{9}+X^{4}+1$	2	0.4777	0.2148
10	$X^{10}+X^{3}+1$	2	0.4950	0.2382
11	$X^{11}+X^{2}+1$	2	0.4898	0.2325
12	$X^{12}+X^{6}+X^{4}+X+1$	4	0.4977	0.2421
13	$X^{13}+X^{4}+X^{3}+X+1$	4	0.4906	0.2469
14	$X^{14}+X^{5}+X^{3}+X+1$	4	0.4975	0.2500
15	$X^{15}+X+1$	2	0.4979	0.2462
16	$X^{16}+X^{5}+X^{3}+X^{2}+1$	4	0.4978	0.2490

## Data Availability

The original contributions presented in the study are included in the article, further inquiries can be directed to the corresponding author.
